# CDK4: A Novel Therapeutic Target for Extramammary Paget’s Disease

**DOI:** 10.3389/fonc.2021.710378

**Published:** 2021-07-29

**Authors:** Hiroki Hashimoto, Yumiko Kaku-Ito, Yoshinao Oda, Takamichi Ito

**Affiliations:** ^1^Department of Dermatology, Graduate School of Medical Sciences, Kyushu University, Fukuoka, Japan; ^2^Department of Anatomic Pathology, Graduate School of Medical Sciences, Kyushu University, Fukuoka, Japan

**Keywords:** extramammary Paget’s disease, CDK4, cyclin D1, prognostic factor, targeted therapy, CDK4/6 inhibitor

## Abstract

**Background:**

The outcome of extramammary Paget’s disease (EMPD) is poor when it progresses to metastasis because of the lack of effective systemic therapies. Recently, CDK4-targeted therapy has attracted attention as a potential therapeutic target for some cancers. The aim of this study was to analyze the impact of CDK4 expression on the survival of patients with EMPD.

**Methods:**

We retrospectively reviewed 110 patients with EMPD. We conducted immunohistochemical analysis of CDK4 and cyclin D1 expression, and assessed the association between their expression and survival.

**Results:**

Most EMPD lesions (108/110, 98.2%) were positive for CDK4 staining and there was a positive correlation between CDK4 expression and cyclin D1 expression (r = 0.54, *p* < 0.001). Tumor thickness (*p* = 0.0003) and the presence of regional lymph node metastasis (*p* = 0.015) were significantly associated with high CDK4 expression. Regarding invasive EMPD, the multivariate analysis did not show the correlation between the expression of CDK4/cyclin D1 and survival outcomes (HR: 3.14, *p* = 0.14).

**Conclusion:**

The overexpression of CDK4 was identified as a major risk factor for disease progression. CDK4-targeted therapy could thus be a novel treatment option for unresectable EMPD.

## Introduction

Extramammary Paget’s disease (EMPD), first reported by Crocker in 1889, is an uncommon skin cancer that shows a propensity to occur in anogenital/axillary areas in the elderly (1–4). In most EMPD patients, the prognosis is good because most tumors of this type remain restricted to the epidermis as *in situ* lesions and are slow-growing (2, 5). However, we sometimes encounter patients with invasive EMPD, which increases the risk of lymph node and distant metastasis, resulting in a poor prognosis (3, 6–10).

Complete surgical resection is the first-line treatment for EMPD in the localized stage. However, it is sometimes difficult to complete appropriate resection because of an unclear tumor border and the invasion of mucosal areas (e.g., anorectum, urethra, vagina), leading to incomplete resection, tumor recurrence, and subsequent tumor progression to metastasis (9, 11–13). Several therapeutic modalities such as radiation therapy, chemotherapy, and molecular-targeted therapy have been reported (4, 5, 14, 15). In particular, targeted therapies using monoclonal antibodies against human epidermal growth factor receptor 2 (HER2) have been applied for metastatic EMPD with some success (16–20). However, their efficacy in treating unresectable EMPD is unsatisfactory. Recently, several genomic profiling analyses using genomic sequencing were conducted in order to determine driver mutations in EMPD (21–23). Despite the progress made, new treatment modalities are required (24–26).

The cyclin D1 cyclin-dependent kinase (CDK)4/6 has been identified as an important factor in several malignant tumors. CDK4/6 controls the cell cycle progression through its interaction with cyclin D1. Clinical trials or preclinical studies on CDK4/6 inhibitors have been implemented for some malignant tumors (27–29). Moreover, CDK4/6 inhibitors are currently widely used for the treatment of breast cancer, and their therapeutic value in this disease context has attracted significant attention (30–33). Regarding EMPD, a recent report (34) suggested that CDK4 and cyclin D1 are overexpressed in EMPD tumor cells. However, the impact of their expression on prognosis has not been elucidated.

In this study, we examined CDK4 expression in 110 clinical EMPD samples using immunohistochemistry in our institution. We also examined the expression of cyclin D1 and analyzed its correlation with CDK4 expression. In addition, we assessed the associations between CDK4/cyclin D1 expression and patient survival.

## Materials and Methods

### Data Collection

We retrospectively collected the data of 110 patients with primary EMPD lesions. These patients were treated at the Department of Dermatology, Kyushu University (Fukuoka, Japan), between January 1997 and December 2018. At least three experienced dermatopathologists confirmed the diagnosis. Cases of secondary EMPD, with direct expansion from cancer of a visceral organ, were excluded. The following patient data were collected: demographic data (sex, age at initial presentation), clinical data (tumor site, primary lesion size), and histopathological data obtained from the surgical specimen (tumor thickness, lymphovascular invasion). Tumor thickness was measured to the second decimal place, as per the latest melanoma classification guidelines of the American Joint Committee on Cancer (35). For patients with two or more primary lesions, we recorded the greatest tumor thickness and total tumor size. Lymph node metastasis was primarily determined by histopathology. Patients who had lymphadenopathy detected by physical examination or imaging studies [ultrasonography, computed tomography (CT), and/or positron emission tomography with computed tomography (PET/CT)] were also considered to have metastasis. Distant metastasis was also determined by imaging studies. The TNM stage was defined in accordance with an EMPD-specific staging system (6). The T category was determined according to tumor thickness and lymphovascular invasion: T0, *in situ* tumor; T1, tumor thickness ≤ 4 mm and no lymphovascular invasion; and T2, tumor thickness > 4 mm or lymphovascular invasion. The N category was categorized as follows: N0, no lymph node metastasis; N1, metastasis involving one lymph node; and N2, metastasis involving two or more lymph nodes. For the M category, M0 indicated no distant metastasis, and M1 indicated distant metastasis.

This retrospective review of our patients was conducted in accordance with the guidelines of the Declaration of Helsinki. This study was approved by the Ethics Committee of Kyushu University Hospital (30–363; November 27, 2018).

### Immunohistochemistory

All formalin-fixed (24 h in 10% buffered formalin), paraffin-embedded EMPD tissues were obtained from the archives of Kyushu University Hospital. Immunohistochemical staining was performed as reported previously with slight modifications (34). Tissue samples were cut into 4 µm sections. For immunostaining, antigen retrieval was performed with Heat Processor Solution pH 9 (Nichirei Biosciences, Tokyo, Japan) for CDK4 and cyclin D1 at 100°C for 40 min. The primary antibody was diluted with Dako REAL Antibody Diluent (s2022; Dako Denmark A/S, Glostrup, Denmark). The sections were incubated with primary rabbit monoclonal antibodies against CDK4 (1:400, 12790; Cell Signaling Technology, Beverly, MA, USA) and cyclin D1 (1:200, 55506; Cell Signaling Technology, Beverly, MA, USA) at 4°C overnight, followed by incubation with N-Histofine Simple Stain MAX-PO MULTI (724152; Nichirei Biosciences) as the secondary antibody for 30 min at room temperature. Immunoreactions were detected using 3,3′-diaminobenzidine tetrahydrochloride (725191; Nichirei Biosciences) as a chromogenic substrate, and specimens were counterstained using hematoxylin. Cytokeratin 7 (CK7) was also stained simultaneously to distinguish EMPD tumor cells. Antigen was retrieved *via* incubation with protease (715231; Nichirei Biosciences) for 5 min followed by mouse anti-human CK7 (prediluted by the supplier, 713481; Nichirei Biosciences) as the primary antibody for 30 min at room temperature. We then incubated sections with N-Histofine Simple Stain AP MULTI (414261; Nichirei Biosciences) as the secondary antibody for 30 min at room temperature. Immunoreactions were detected using FastRed II (415261; Nichirei Biosciences) as a chromogen, and specimens were counterstained using hematoxylin.

### Evaluation of CDK4/Cyclin D Immunohistochemical Staining

The immunohistochemical results were evaluated by a semiquantitative approach using the H-score (36, 37). The intensity of staining was graded as follows: no staining (0), weakly positive (1+), moderately positive (2+), and strongly positive (3+) (**Supplementary Figure 1**). Neither CDK4 nor cyclin D1 was stained in the normal epidermis. The staining of CDK4 and cyclin D1 in EMPD was generally homogeneous. The H-scores of CDK4 and cyclin D1 were calculated as the percentage of positive cells (0%–100%) multiplied by the staining intensity (0–3+), with the final score ranging from 0 to 300. The H-scores were calculated by counting tumor cells in three random high-power fields (×200). Two independent dermatologists (H.H. and T.I.) who were blinded to the clinical information assessed the sections. Images were taken using an ECLIPSE 80i microscope (Nikon, Tokyo, Japan). The sample was divided into two groups based on the mean H-score, and patient background and prognosis were compared.

### Treatment and Follow-up

All patients underwent surgical excision for their primary lesions, basically with wide margins (1.0–5.0 cm). For regional lymph node metastasis or distant metastasis, the patients underwent complete lymph node dissection, systemic chemotherapy, and/or radiation therapy alone or in an appropriate combination. They were monitored by physical examination every 3–6 months and imaging (ultrasonography, chest X-ray, and/or CT). Survival data, including the duration of survival and cause of death, were recorded. The median follow-up period was 85.1 months (range: 2.0–225.8 months). By the last follow-up, 79 patients were alive, 13 had died of EMPD, and 18 had died of other causes.

### Statistical Analysis

All statistical analyses were performed using JMP version 14.2 (SAS Institute, Cary, NC, USA). The χ^2^ test or Fisher’s exact test was used to analyze categorical variables, whereas the Mann–Whitney U test was used to analyze continuous variables. Correlations between the levels of CDK4 and cyclin D1 staining were assessed by Pearson’s correlation coefficient analysis. We used the Kaplan-Meier method to evaluate disease-specific survival (DSS), and we compared survival curves using the log-rank test. DSS was calculated from the date of the first histological examination to the date of death due to EMPD or the last follow-up prior to March 31, 2021. Data for patients who did not die were censored on March 31, 2021. Data for patients who died of other causes were censored at the time of death. The associations of clinical and histopathological factors with DSS were determined using a multivariate Cox proportional hazards regression model. P-values less than 0.05 were regarded as statistically significant.

## Results

### Clinicopathological Data of the Study Cohort

The demographic and clinical data of the 110 patients with primary EMPD are shown in **Table 1**. All patients were Japanese, with a mean age of 72.6 years (range: 42–91 years). There were 70 male patients (63.6%) and 40 female patients (36.4%). Tumors were predominantly localized in the genital area (84.5%), followed by the perianal area (3.6%) and the axillary area (3.6%). Multiple lesions or tumors spreading over two areas were seen in nine patients (8.2%), including eight patients with lesions in the perianal area. There were 53 patients (48.2%) with small primary lesions (< 25 cm^2^) and 57 (51.8%) with large lesions (≥ 25 cm^2^). A total of 62 patients (56.4%) had tumors *in situ* and the remaining 48 patients (43.6%) had invasive tumors. Tumor thickness was stratified as ≤ 1 mm, 1–4 mm, or > 4 mm for invasive tumors. There were 15 patients (13.6%) with tumors ≤ 1 mm, 22 (20.0%) with tumors 1–4 mm, and 11 (10.0%) with tumors > 4 mm. Lymphovascular invasion was observed in nine patients (8.2%). Regional lymph node metastasis was found in 14 patients (12.7%). Five patients (4.5%) had one metastatic lymph node, and nine (8.2%) had two or more. Distant metastasis was observed in five patients (4.5%).

**Table 1:** Demographics and clinical data of all 110 patients.

Parameter	n (%)
Sex	
Male	70 (63.6)
Female	40 (36.4)
Age (years)	
Mean ± SD	72.6 ± 9.4
Median (range)	72 (42-91)
Tumor site	
Genital area only	93 (84.5)
Perianal area only	4 (3.6)
Axillary area only	4 (3.6)
Genital + perianal areas	6 (5.5)
Genital + axillary areas	1 (0.9)
Perianal + axillary areas	1 (0.9)
Genital + perianal + axillary areas	1 (0.9)
Primary lesion size (cm^2^)	
< 25	53 (48.2)
≥ 25	57 (51.8)
TT (mm)	
In situ	62 (56.4)
≤ 1	15 (13.6)
1–4	22 (20.0)
> 4	11 (10.0)
Lymphovascular invasion	
Present	9 (8.2)
Absent	101 (91.8)
Metastasis	
Regional LN metastasis	
N0	96 (87.3)
N1	5 (4.5)
N2	9 (8.2)
Distant metastasis	
M0	105 (95.5)
M1	5 (4.5)

SD, standard deviation; TT, tumor thickness; LN, lymph node.

### CDK4/Cyclin D1 Expression in EMPD

**Figure 1:**
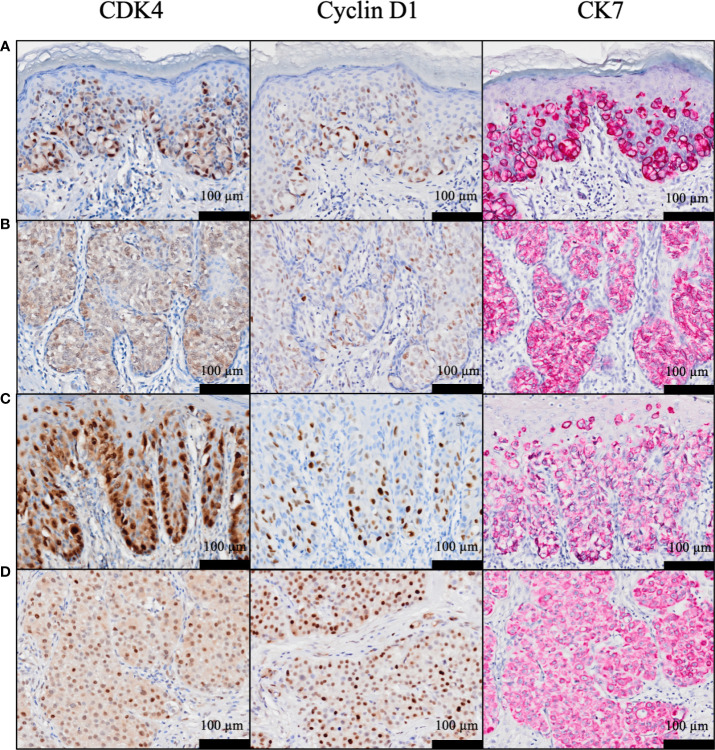
**(A–D)** Representative histopathological images of CDK4, cyclin D1, and cytokeratin 7 staining in EMPD. H-scores for CDK4 were: **(A)** 153, **(B)** 121, **(C)** 185, and **(D)** 200, and H-scores for cyclin D1 were: **(A)** 121, **(B)** 90, **(C)** 106, and **(D)** 195.

**Figure 2:**
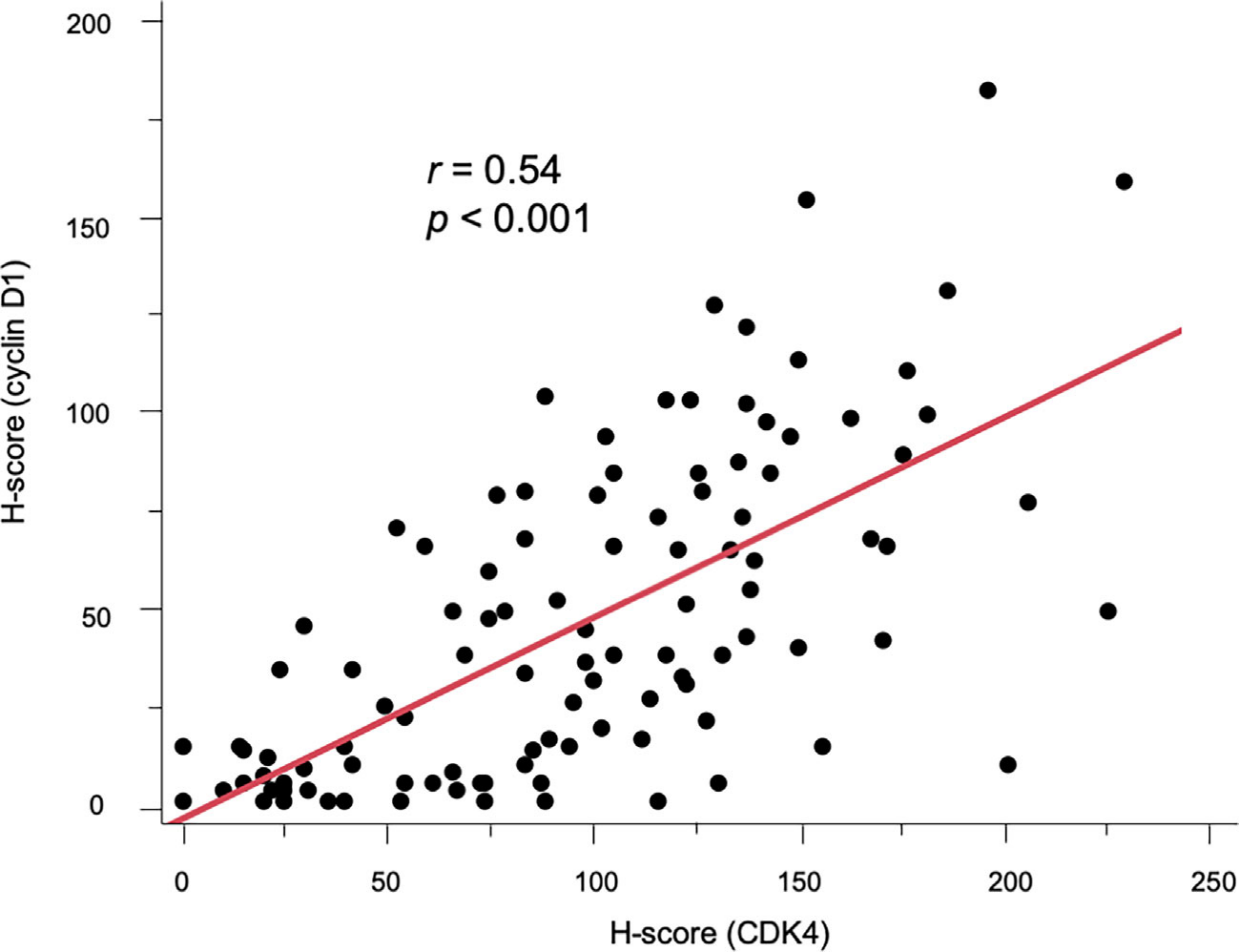
Scatter diagram showing the positive correlation between CDK4 and cyclin D1 expression (r = 0.54, *p* < 0.001).

As previously reported (34), neither CDK4 nor cyclin D1 was observed in the epidermis. CK7 was stained simultaneously to distinguish EMPD tumor cells. Positive CK7 staining was indicated by a red color. Representative images of CDK4, cyclin D1, and CK7 staining in EMPD lesions are presented in **Figure 1**. The majority of the tumor cells were positive for CDK4 staining and cyclin D1 staining in the nuclei. Cytoplasmic staining was observed in association with nuclear staining. Almost all EMPD samples (108/110, 98.2%) were positive for CDK4 staining (staining was defined as positive when at least some of the tumor cells were stained), and 98 of 110 (89.1%) EMPD samples were positive for cyclin D1 staining at various staining intensities. Only one (0.9%) EMPD sample showed negative staining (H-score: 0) for both CDK4 and cyclin D1, which involved EMPD in situ. The mean H-score of CDK4 staining was 97.7 and the median score was 96.5 (range: 0–234). The mean H-score of cyclin D1 staining was 47.9 and the median score was 39 (range: 0–195). **Figure 2** shows the correlation between CDK4 and cyclin D1 expression. There was a positive correlation between these staining levels (r = 0.54, *p* < 0.001). We divided the sample into two sets of two groups based on the mean H-scores: CDK4-low (H-score ≤ 97) and CDK4-high (H-score > 97), and cyclin D1-low (H-score ≤ 47) and cyclin D1-high (H-score > 47).

### Association of CDK4/Cyclin D1 With Clinicopathological Factors

**Table 2** presents the associations between immunohistochemical CDK4/cyclin D1 expression and clinicopathological factors. In total, 54 (49.1%) and 56 patients (50.9%) were categorized into the CDK4-high and CDK4-low groups, respectively. Among these factors, tumor thickness (*p* = 0.0003) and the presence of regional lymph node metastasis (*p* = 0.015) were significantly associated with high CDK4 expression. Regarding the expression of cyclin D1, 47 (42.7%) and 63 patients (57.3%) were categorized into the cyclin D1-high and cyclin D1-low groups, respectively. No factors were significantly associated with the expression of cyclin D1.

**Table 2:** Factors associated with CDK4 and cyclin D1 expression.

Parameters	CDK4 Expression		*p*-value*	Cyclin D1 Expression		*p*-value*
	Low (n = 56)	High (n = 54)		Low (n = 63)	High (n = 47)	
Sex						
Male	34 (60.7%)	36 (66.7%)	0.56	37 (58.7%)	33 (70.2%)	0.23
Female	22 (39.3%)	18 (33.3%)		26 (41.3%)	14 (29.8%)
Age (year)						
Mean ± SD	72.0 ± 9.5	73.1 ± 9.3	0.74	72.3 ± 9.4	72.9 ± 9.4	0.90
Tumor site						
Perianal area	5 (8.9%)	7 (13.0%)	0.55	8 (12.7%)	4 (8.5%)	0.55
Other areas	51 (91.1%)	47 (87.0%)		55 (87.3%)	43 (91.5%)	
Primary lesion size (cm^2^)						
< 25	29 (51.8%)	24 (44.4%)	0.45	33 (52.4%)	20 (42.6%)	0.34
≥ 25	27 (48.2%)	30 (55.6%)		30 (47.6%)	27 (57.4%)	
TT (mm)						
In situ	42 (75.0%)	20 (37.0%)	**0.0003** ^†^	39 (61.9%)	23 (48.9%)	0.23^‡^
≤ 4	11 (19.6%)	26 (48.2%)		20 (31.7%)	17 (36.2%)	
> 4	3 (5.4%)	8 (14.8%)		4 (6.4%)	7 (14.9%)	
Regional LN metastasis						
Present	3 (5.4%)	11 (20.4%)	**0.015**	7 (11.1%)	7 (14.9%)	0.58
Absent	53 (94.6%)	43 (79.6%)		56 (88.9%)	40 (85.1%)	
TNM stage						
0, I, II	52 (92.9%)	43 (79.6%)	0.054	55 (87.3%)	40 (85.1%)	0.78
III, IV	4 (7.1%)	11 (20.4%)		8 (12.7%)	7 (14.9%)	
FU (month)						
Mean ± SD	96.3 ± 45.7	78.8 ± 54.0	**0.043**	91.0 ± 46.7	83.3 ± 55.4	0.26
Median (range)	100.0 (4.9–189.8)	66.3 (2.0–225.8)		91.9 (4.9–178.2)	72.9 (2.0–225.8)	

Significant values are shown in boldface.

*Mann-Whitney U tests were used for continuous variables, and χ^2^ or Fisher’s exact tests were used for categorical variables.

^†^In situ vs. ≤ 4 mm, p = 0.0004; in situ vs. > 4 mm, p = 0.017; ≤ 4 mm vs. > 4 mm, p = 1.00.

^‡^In situ vs. ≤ 4 mm, p = 0.41; in situ vs. > 4 mm, p = 0.18; ≤ 4 mm vs. > 4 mm, p = 0.49.

CDK, cyclin-dependent kinase; SD, standard deviation; TT, tumor thickness; LN, lymph node; TNM, tumor, node, metastasis; FU, follow-up period.

### Prognostic Impact of CDK4 and Cyclin D1 Expression

**Figure 3:**
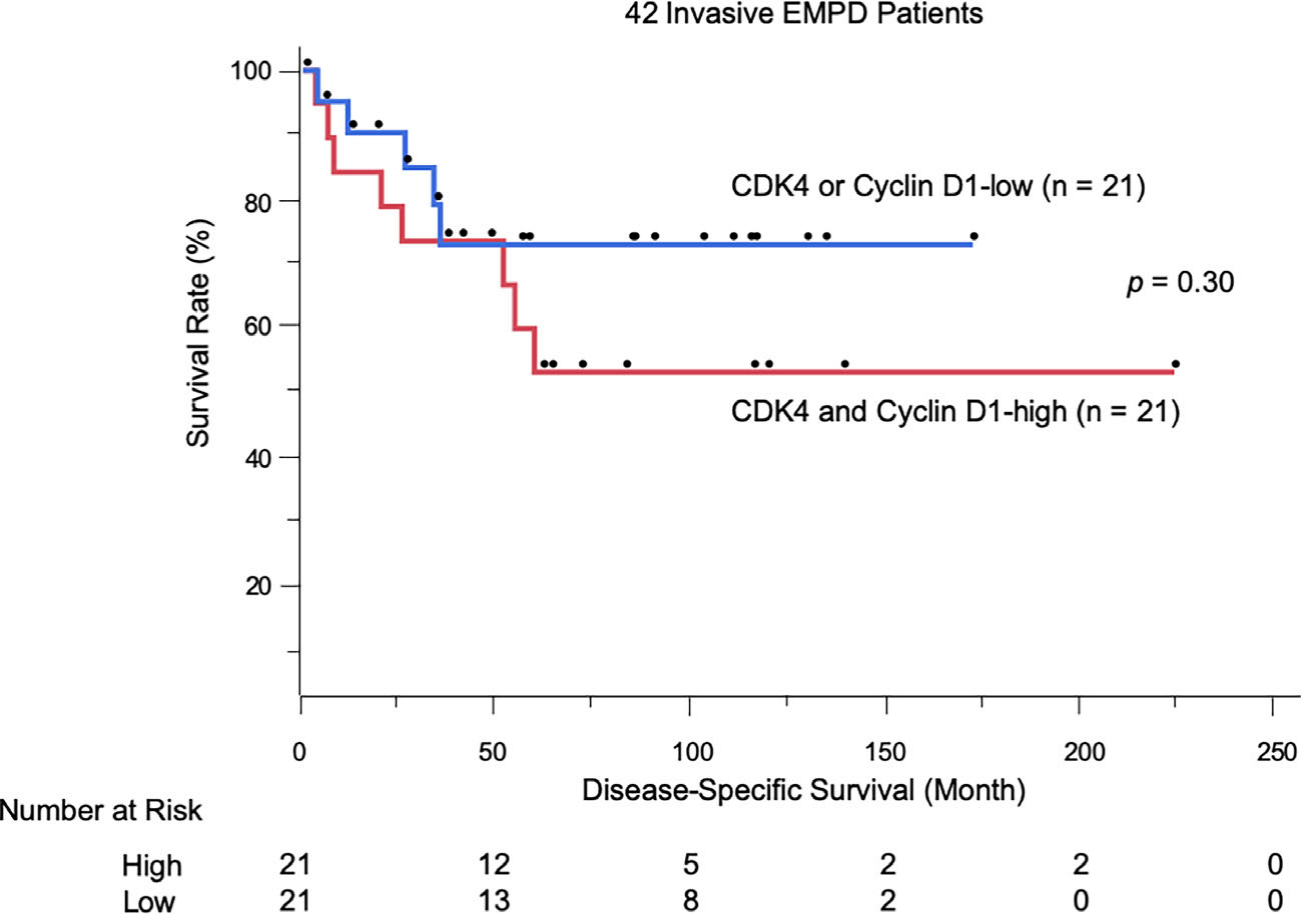
Kaplan-Meier disease-specific survival curves of 42 patients with invasive EMPD stratified by CDK4/cyclin D1 expression.

We evaluated the possible clinical and histopathological factors associated with DSS by using a multivariate Cox proportional hazards regression model. First, the survival analysis was performed in patients with invasive EMPD. Since six patients with invasive EMPD who had died of other causes were excluded from the survival analysis, 42 patients were included in the analysis. The following factors were included as explanatory variables: sex, age, tumor site, tumor size, TNM stage, and CDK4/cyclin D1 expression. Since there was a strong correlation between the expression of CDK4 and that of cyclin D1 (r = 0.54, *p* < 0.001), multivariate analysis was performed under the condition that the expression levels of both of these molecules were high. Tumor thickness and regional lymph node metastasis were excluded from the model because they strongly correlated with CDK4 expression and TNM stage. The results are listed in **Table 3**. The results of univariate analysis revealed that TNM advanced stage was statistically significant factors for poor survival (HR: 11.84, *p* = 0.0002). Multivariate analysis confirmed the associations of TNM advanced stage with DSS (HR: 19.24, *p* < 0.0001). The overexpression of both CDK4 and cyclin D1 was not a statistically significant factor for poor survival (HR: 3.14, *p* = 0.14), possibly due to the insufficient number of patients with invasive EMPD. The Kaplan-Meier curves of patients stratified by CDK4/cyclin D1 expression are shown in **Figure 3**.

**Table 3:** Multivariate Cox proportional hazards analysis for disease-specific survival in 42 patients with invasive EMPD.

Variable	Univariate analysis			Multivariate analysis		
	HR	95% CI	*p*-value	HR	95% CI	*p*-value
Sex, male	0.47	0.16-1.40	0.17	0.35	0.065–1.85	0.22
Age (year)^†^	1.01	0.96-1.07	0.69	1.04	0.97–1.12	0.29
Perianal lesion	1.47	0.40-5.35	0.56	1.62	0.22–11.74	0.63
Tumor size, >25 cm^2^	1.28	0.43-3.84	0.65	0.51	0.12-2.16	0.36
TNM stage, III or IV	11.84	3.18-44.08	**0.0002**	19.24	4.38-84.61	**< 0.0001**
CDK4 and cyclin D1 expression, high	1.78	0.58-5.45	0.31	3.14	0.68-14.57	0.14

Significant values are shown in boldface.

^†^Continuous variable.

CDK, cyclin-dependent kinase; HR, hazard ratio; CI, confidence interval; TNM, tumor, node, metastasis.

The additional analysis was performed on 92 patients with EMPD, including the cases of tumor *in situ* and excluding those who had died of other causes. The results of univariate analysis revealed that TNM advanced stage and overexpression of both CDK4 and cyclin D1 were statistically significant factors for poor survival. Multivariate analysis confirmed the associations of TNM advanced stage (HR: 43.08, *p* < 0.0001), and overexpression of both CDK4 and cyclin D1 (HR: 4.76, *p* = 0.033) with DSS. The results of multivariate analysis and Kaplan-Meier curves of EMPD patients stratified by CDK4 expression and cyclin D1 expression are available in **Supplementary Table 1** and **Supplementary Figures 2**, **3**.

## Discussion

The cyclin D1-CDK4/6 inhibitor of the CDK4 (INK4)- retinoblastoma (Rb) signaling pathway regulates cellular proliferation by controlling the first gap (G_1_)- to DNA synthesis (S)-phase cell cycle checkpoint. Dysregulation of this pathway is frequently observed in malignant tumors and contributes to cell cycle progression and continued growth (27, 38). For example, overexpression of at least one cyclin D1-CDK4/6-INK4-Rb pathway component occurs in most cases of breast cancer (39), melanoma (40), head and neck squamous cell carcinoma (41), and liposarcoma (42, 43). Abnormal dysregulation of the CDK4/6-cyclin D1 complex is a hallmark of cancer.

Although the overexpression of CDK4 and cyclin D1 is also observed in EMPD tumor cells (34, 44, 45), the correlation between their expression and patients’ prognosis has not been elucidated. In this study, we retrospectively reviewed 110 patients with EMPD, and assessed the associations between CDK4/cyclin D1 expression and survival. Almost all EMPD lesions (108/110, 98.2%) exhibited CDK4 expression. Notably, high CDK4 expression in EMPD was significantly associated with greater tumor thickness and the presence of lymph node metastasis, which are known as prognostic factors (46, 47). Our results imply that the overexpression of CDK4 and cyclin D1 accelerates the progression of EMPD. In the multivariate analysis on patients with invasive EMPD, the overexpression of both CDK4/cyclin D1 was not associated with poor survival outcomes (HR: 3.14, *p* = 0.14), possibly due to the insufficient number of patients with invasive EMPD. The analysis including the cases of tumor *in situ* revealed that the overexpression of both CDK4 and cyclin D1 in EMPD tumor cells was significantly correlated with worse DSS (HR: 4.76, *p* = 0.033).

CDK4 staining was positive in all advanced EMPD lesions, suggesting that CDK4-targeted therapy should be effective against advanced EMPD. In vitro and *in vivo* investigations revealed that CDK4/6 inhibitors suppress the proliferation of many different malignant tumor cells (40, 42, 48). Owing to the significance of CDK4/6 kinases in the regulation of cell proliferation, CDK4/6 inhibitors have undergone clinical trials for several malignant tumors. For example, in a phase III trial of patients with stage IV non-small cell lung cancer harboring KRAS mutations, abemaciclib demonstrated improvement in progression-free survival compared with erlotinib (49), and a phase III trial of patients with recurrent stage IV squamous cell lung cancer is ongoing (NCT02154490/Lung-MAP). As CDK4-targeted therapy, CDK4/6 inhibitors (e.g., abemaciclib, palbociclib, ribociclib) are currently widely used for the treatment of breast cancer, and their therapeutic value in this disease context has attracted significant attention (30–33). As in these malignant tumors, CDK4/6 inhibitors are also anticipated to be valuable for unresectable EMPD.

Currently, no consensus regarding the optimal chemotherapy or molecular-targeted therapy for unresectable EMPD has been reached because of its rarity and the lack of clinical trials. We previously evaluated the efficacy of conventional chemotherapy for metastatic EMPD and found that conventional chemotherapy improved progression-free survival, but not overall survival (15). Conventional chemotherapy also has a relatively high incidence of adverse events. Since targeted therapies have also recently been shown to be effective for metastatic EMPD (16–20), they may become a mainstay for the treatment of EMPD in the future. The identification of molecules in EMPD tumor cells that correlate with prognosis, such as CDK4, should lead to the establishment of novel therapeutic approaches.

This study was limited by the inherent potential bias of retrospective studies. In addition, the number of patients with metastatic EMPD was inadequate (15 of 110 patients, 13.6%) and more than half patients (62 of 110 patients, 56.4%) had tumors in situ. The findings suggested that targeted therapy is applicable in unresectable or metastatic EMPD. However, to obtain further support for our findings, further data accumulation should be desired.

## Conclusion

We retrospectively reviewed 110 patients with EMPD. Most EMPD lesions (108/110, 98.2%) were positive for CDK4 staining and there was a positive correlation between CDK4 and cyclin D1 expression. The overexpression of CDK4 and cyclin D1 in combination was also associated with advanced tumor. CDK4-targeted therapy may be effective against advanced EMPD.

## Data Availability Statement

The raw data supporting the conclusions of this article will be made available by the authors, without undue reservation.

## Ethics Statement

The studies involving human participants were reviewed and approved by Kyushu University Hospital. The patients/participants provided their written informed consent to participate in this study.

## Author Contributions

HH and TI participated in manuscript preparation. TI designed the methodology. HH participated in data analysis and figure preparation. HH and YK-I collected the detailed information of the patients. TI and YO reviewed and revised the manuscript. All authors contributed to the article and approved the submitted version.

## Funding

This work was supported by grants from the Takeda Science Foundation and JSPS KAKENHI (Grant Number 19K16867).

## Conflict of Interest

The authors declare that the research was conducted in the absence of any commercial or financial relationships that could be construed as a potential conflict of interest.

## Publisher’s Note

All claims expressed in this article are solely those of the authors and do not necessarily represent those of their affiliated organizations, or those of the publisher, the editors and the reviewers. Any product that may be evaluated in this article, or claim that may be made by its manufacturer, is not guaranteed or endorsed by the publisher.

## Abbreviations

EMPD, extramammary Paget’s disease; CDK, cyclin-dependent kinase; CK, Cytokeratin; Rb, retinoblastoma; TNM, tumor, node, and metastasis; DSS, disease-specific survival; SLNB, sentinel lymph node biopsy; CLND, completion lymph node dissection.
