# Supplementary figures and images for: CDK4: A Novel Therapeutic Target for Extramammary Paget’s Disease
Source: Front Oncol. 2021 Jul 29;11:710378. doi: 10.3389/fonc.2021.710378 (PMC8358779; doi:10.3389/fonc.2021.710378)

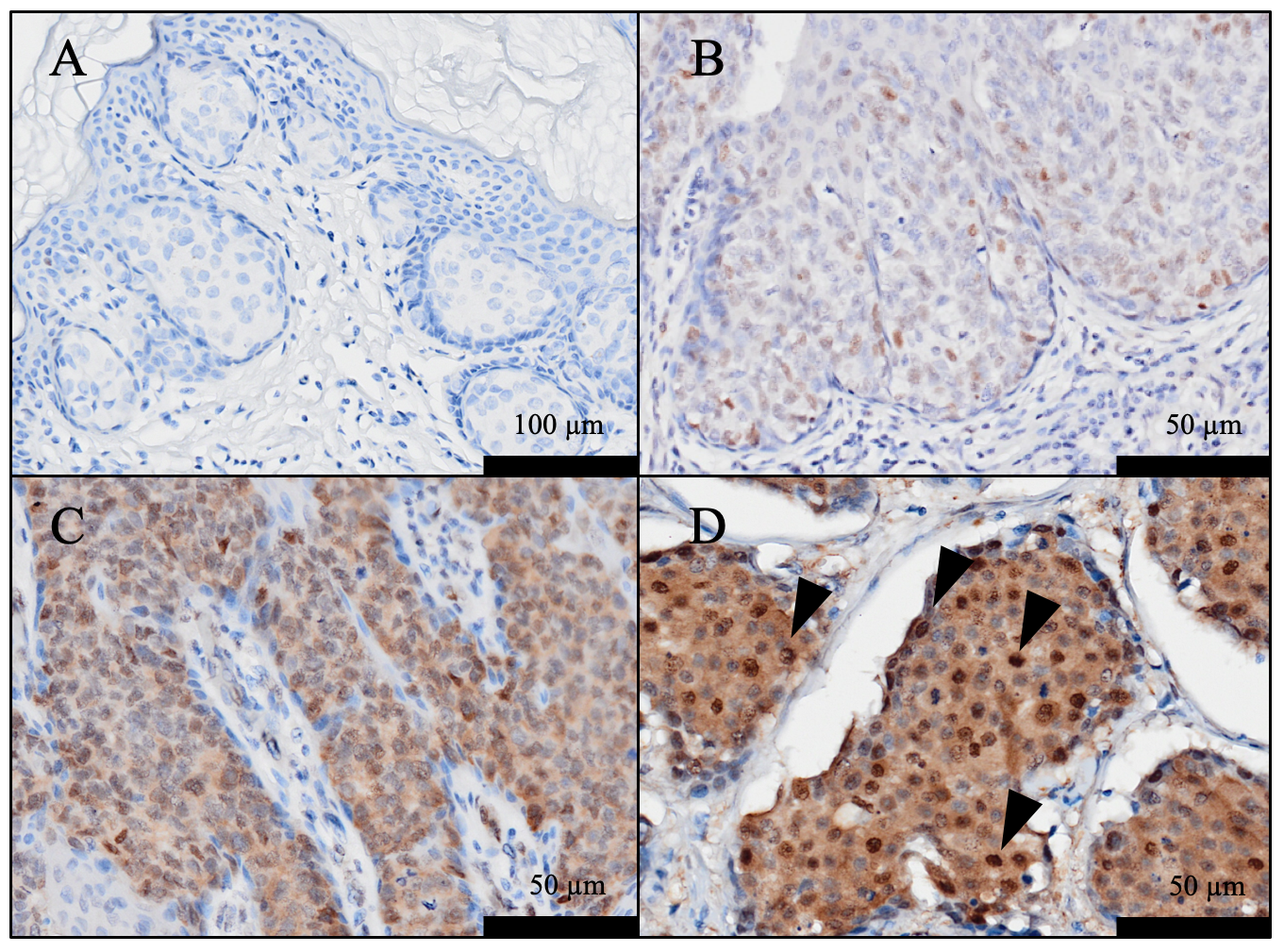

Supplement: Supplementary file 1 [file Image_1.tiff]

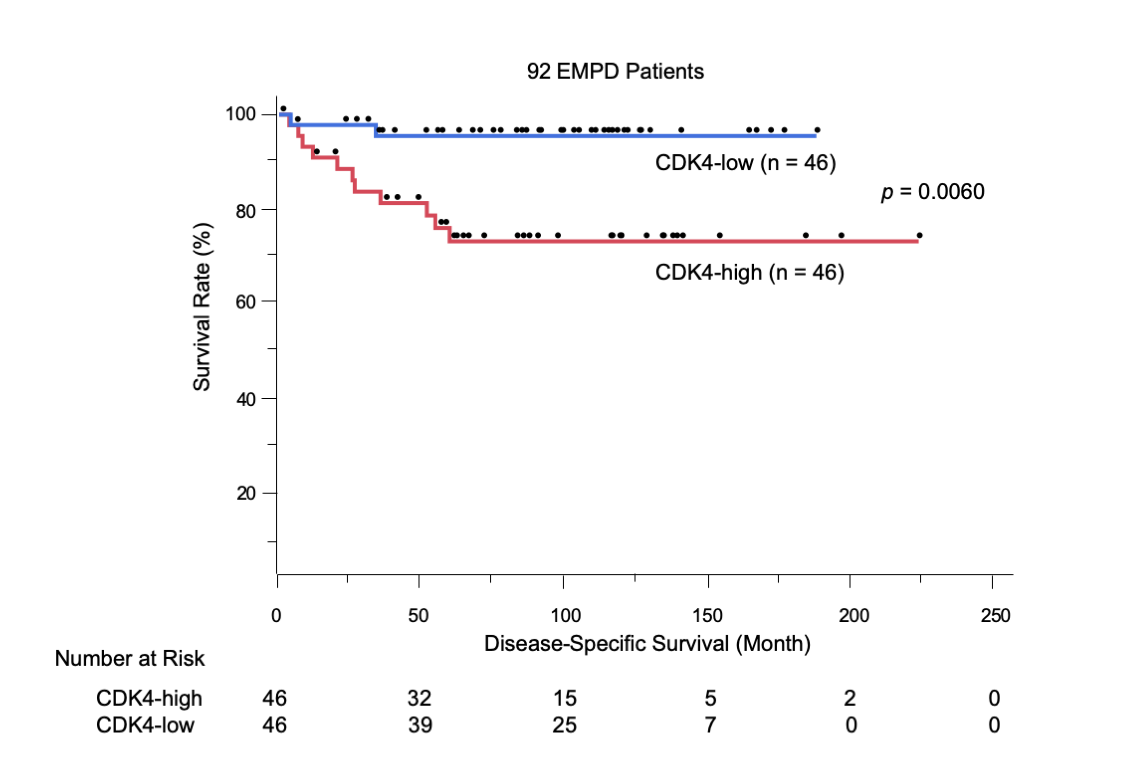

Supplement: Supplementary file 2 [file Image_2.tiff]

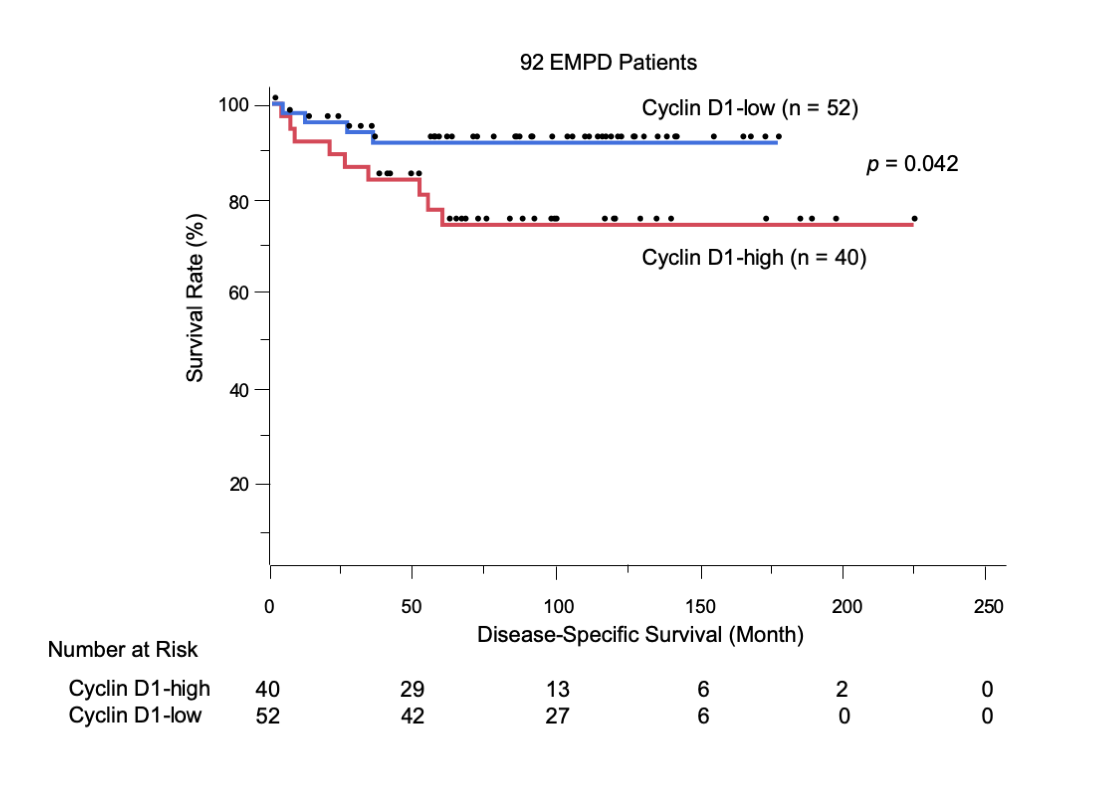

Supplement: Supplementary file 3 [file Image_3.tiff]
