# Supplementary material for: CDK4: A Novel Therapeutic Target for Extramammary Paget’s Disease
Source: Front Oncol. 2021 Jul 29;11:710378. doi: 10.3389/fonc.2021.710378 (PMC8358779; doi:10.3389/fonc.2021.710378)
Supplement: Supplementary file 4 [file DataSheet_1.pdf]

Supplementary Material

1 Supplementary Figures

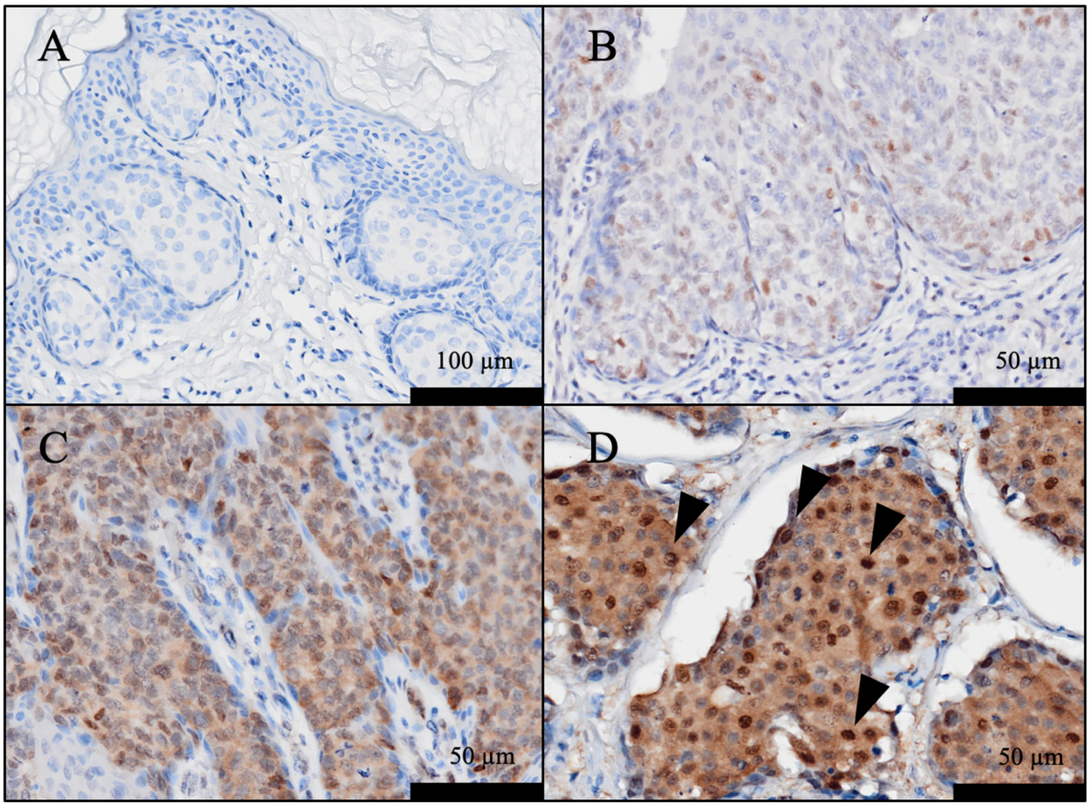

Supplementary Figure 1. Staining intensity of CDK4 and cyclin D1 antibody. Positive signals are indicated by a brown color. (A) Negative staining (0), (B) weakly positive staining (1+), (C) moderately positive staining (2+), and (D) strongly positive staining (arrowheads) (3+).

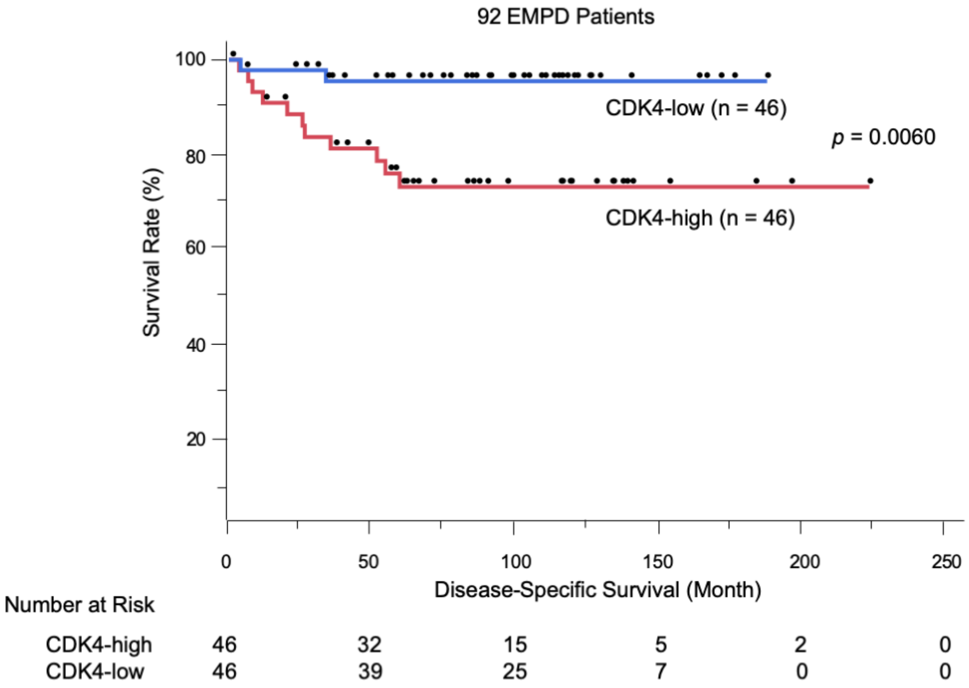

Supplementary Figure 2. Kaplan-Meier disease-specific survival curves of patients with 92 EMPD stratified by CDK4 expression. CDK4-high patients had significantly shortened survival (p = 0.0060).

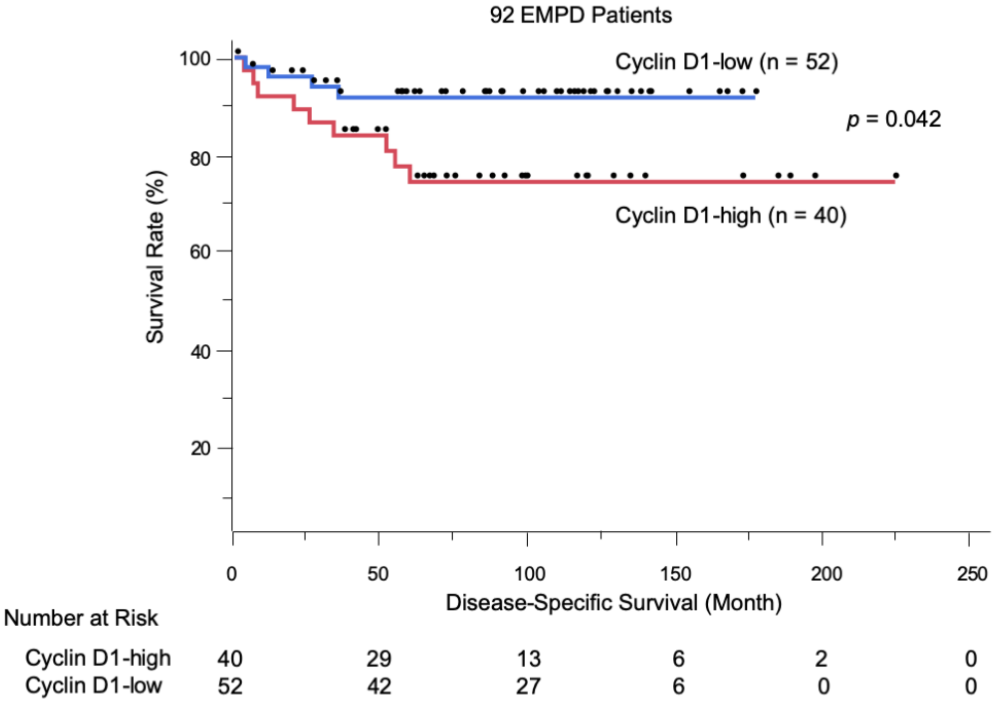

Supplementary Figure 3. Kaplan-Meier disease-specific survival curves of patients with 92 EMPD stratified by cyclin D1 expression. Cyclin D1-high patients had significantly shortened survival ($p = 0.042$).

2 Supplementary Table

Supplementary Table 1. Multivariate Cox proportional hazards analysis for disease-specific survival in 92 patients with EMPD

Variable	Univariate analysis			Multivariate analysis		
	HR	95% CI	*p*-value	HR	95% CI	*p*-value
Sex, male	0.66	0.22-1.97	0.46	0.44	0.078–2.43	0.34
Age (year)†	1.03	0.97-1.10	0.42	1.05	0.98–1.13	0.14
Perianal lesion	2.35	0.65-8.54	0.20	2.30	0.33–16.07	0.40
Tumor size, >25 cm^2	0.83	0.28-2.48	0.75	0.36	0.083-1.53	0.16
TNM stage, III or IV	36.99	9.88-138.56	**< 0.0001**	43.08	10.06-184.55	**< 0.0001**
CDK4 and cyclin D1 expression, high	4.00	1.31-12.22	**0.015**	4.76	1.14-19.91	**0.033**

Significant values are shown in boldface.

†Continuous variable

CDK, cyclin-dependent kinase; HR, hazard ratio; CI, confidence interval; TNM, tumor, node, metastasis.
